# Pediatric Germ Cell Tumors; A 10-year Experience

**Published:** 2014-07-25

**Authors:** Ahmad Khaleghnejad-Tabari, Alireza Mirshemirani, Mohsen Rouzrokh, Leily Mohajerzadeh, Nasibeh Khaleghnejad-Tabari, Shaghayegh Hasas-Yeganeh

**Affiliations:** 1 Pediatric Surgery Research Center, Shahid Beheshti University of Medical Sciences, Tehran; 2 Digestive Disease Research Center, Tehran University of Medical Sciences, Tehran, Iran

**Keywords:** Germ Cell Tumor, Yolk Sac Tumor, Teratoma, Malignant Tumor

## Abstract

***Objective:*** The aim of this study was to evaluate the outcome of germ cell tumors in patients admitted to our center during a ten year period.

***Methods:*** In a retrospective descriptive study, patients with the pathological diagnosis of germ cell tumor (GCT) were included. All records were evaluated and patients followed by personal visit in clinic or phone call. Data regarding age, sex, tumor site, bio-chemical assay, pathology, treatment and outcomes were gathered. For qualitative variables we computed frequency and percentage and for quantitative variables, mean and standard deviation. Survival analysis was performed using Kaplan-Meier. All statistical analyses were performed by SPSS version16.0.

***Findings*** ***:*** Forty four patients consisted of 32 girls (72.7%) and 12 boys (27.3%). Their median age was 23 months. The most common pathological tumor types were 18 (40.9%) mature teratomas and 14 (31.8%) yolk sac tumors. Extra gonadal tumors were more prevalent (32 cases) and consisted of 21 (47.7%) sacrcoccygeal, 7 (15.9%) retroperitoneal, 2 (4.4%) mediastinal and 2 (4.4%) cervical tumors. In gonadal tumors 9 patients had ovarian and 3 patients testicular involvement. Staging at the time of diagnosis revealed stage one in 23 (52.3%) cases. All patients were treated surgically and the most common procedure was total resection in 41 (93.2%) patients. Fifteen (34.1%) patients received chemotherapy. In follow-up 31 (77.5%) patients were in complete remission, 9 (22.5%) had died, and 4 cases did not appear to follow-up visits. The median survival was 16 months (IQR 4-49 months). The highest mortality rate was found in patients with yolk sac tumors (8 of 13 cases).

***Conclusion:*** The patients with extra-gonadal GCT and a high AFP level have the worst prognosis and lower survival rate**. **Combination of surgery and chemotherapy can lead to a better prognosis.

## Introduction

Germ cell tumors (GCT) are classified as malignant or benign tumors^[^^1^^]^. They consist of a broad variety of neoplasms that all originate from primordial germ cells. Although all of them have the same origin; but are different in pathological behavior and clinical presentation. These types of tumors are not common in children and comprise only 4 percent of childhood cancer^[^^1^^]^. The most common types of GCT are: teratomas, germinomas, endodermal sinus tumor or yolk sac tumors (YSTs)^[^^2^^]^, choriocarcinoma and embryonal carcinoma. YSTs are the most prevalent malignant tumors of the gonads in children^[^^1^^]^. Despite their misleading name which appears to be in association with their anatomic place in the gonadal glands, most of them are extragonadal^[^^3^^]^. Our current knowledge regarding GCT in Iran is incomplete and since our center is a referral center we decided to design a study in order to gather demographic information of germ cell tumors and their treatment outcomes.

## Subjects and Methods

**Study population: **All patients with the pathological diagnosis germ cell tumor (GCT) who were treated in Mofid Children’s Hospital during January 1999 to January 2009 were included. Data regarding age, sex, tumor site, bio-chemical assay, pathology, treatment and outcomes were gathered.

**Patient Treatment and Follow up: **All patients underwent surgery in our center. Patient’s follow-up was carried out either in person in the clinic or by phone. 

**Statistical analysis: **It is a retrospective descriptive study. For qualitative variables we computed frequency and percentage and for quantitative variables, mean and standard deviation. Survival analysis was performed using Kaplan-Meier. All the statistical analyses were performed by SPSS version16.0.

## Findings

Forty four patients entered our study, of which 32 (72.7%) were girls and 12 (27.3%) boys; with a median age of 23 months. The pathological types are shown in Table 1. We had both gonadal and extra gonadal tumors in our patients with extra gonadal tumors being the more prevalent (72.72%). The anatomic distributions of the tumors are shown in Table2. The most prevalent stage at the time of diagnosis was stage 1 and in 5 cases the staging was unknown. Staging of the tumor at the time of diagnosis is summarized in Table 3. AFP and BHCG was measured in all patients.

**Table 1:** Differentiation of pathology type

**Pathologic type**	**Frequency (%)**
**Mature teratoma (benign)**	18 (40.9)
**Immature teratoma**	6 (13.6)
**Yolk sac tumor**	14 (31.8)
**Mixed tumors**	3 (6.8)
**Malignant teratoma**	1 (2.3)
**Dysgerminoma**	1 (2.3)
**Choriocarcinoma**	1 (2.3)
**Total**	44 (100)

 AFP was elevated in 14 cases of yolk sac tumor and one patient of immature teratoma. Also BHCG was high in one case of choriocarcinoma. Those patients who had very high level of AFP (3 times more than the upper limit of normal) died. All patients were treated surgically: total resection in 41 (93.2%) cases, partial resection in 1 (2.3%) case and open biopsy in 2 (4.5%) cases. Fifteen (34.1%) patients received chemotherapy. Follow up results in our patients demonstrated that we had 31 (77.5%) cases with complete remission and 9 (22.5%) deaths; 4 patients were lost to follow up. The median survival in our study was 16 months (IQR 4-49 months).

**Table 2:** Gonadal and extra-gonadal tumor locations

**Sex/ Site **	**Gonadal**	**Extra Gonadal**	**Total**
**Female **	9 (Ovary)	15 (Sacrococcygeal) 5 (Abdomen and Retroperitoneal) 2 (Mediastinum) 1 (Head and Neck)	32 (72.72%)
**Male **	3 (Testis)	6 (Sacrococcygeal) 2 (Abdomen and Retroperitoneal) 1 (Head and Neck)	12 (27.27%)
**Total **	12 (27.27%)	32 (72.72%)	44 (100%)

**Table 3:** Staging of the tumor at the time of diagnosis

**Stage**	**Frequency (%)**
**1**	23 (52.3)
**2**	3 (6.8)
**3**	2 (4.5)
**4**	11 (25)
**Total**	39 (86.1)

**Fig. 1:**
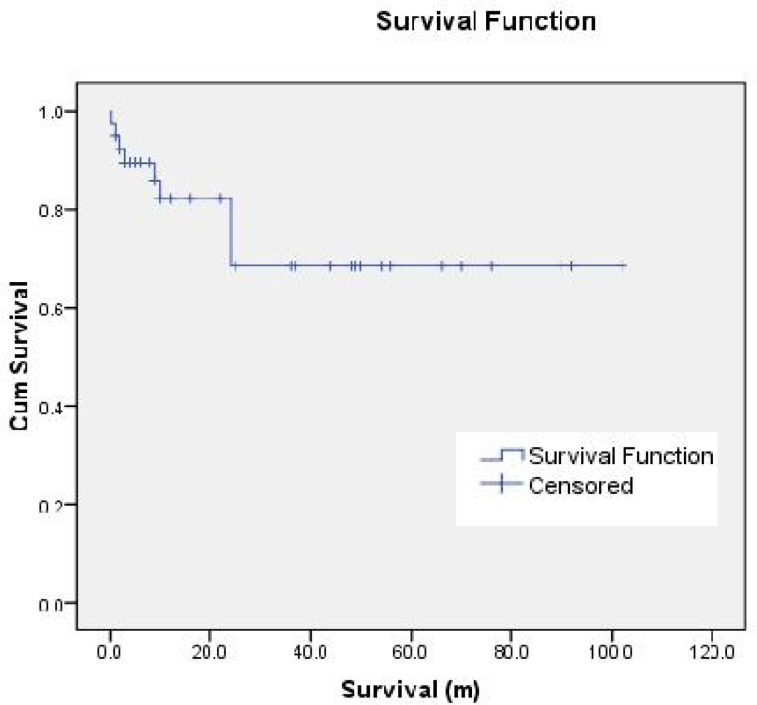
Kaplan–Meier survival plot of the 40 patients after surgery for pediatric germ cell tumors

The Kaplan-Meier survival plot of the patients is shown in Fig 1. The highest mortality rate was found in patients with yolk sac tumors (8 of 13 cases). One other case of mortality was found in a patient with immature teratoma (1 of 6 cases).

## Discussion

GCTs are a rare and diverse group of heterogeneous tumors that include both benign and malignant histologies^[^^4^^]^. Since they are rare, conducting a retrospective research seemed the best policy for gathering information on these tumors in our country and paving the way for future studies.

**Pathologic type: **In our study there were a total of 44 patients, and the most common pathologic type was mature teratoma (41%), in a review by Zachary Horton et al teratomas are considered to be the most prevalent type of germ cell tumors in children^[^^5^^]^. In a study by Billmire and colleagues, which was carried out on 131 children with primary ovarian tumor the most common histology was reported to be teratoma + EST (endodermal sinus tumor ) and teratoma +other malignant elements^[^^6^^]^. Billmire et al. in another study on 26 patients with malignant retroperitoneal and abdominal germ cell tumors reported that the most common pathologic type was pure yolk sac tumor^[^^7^^]^.

**Location: **We had 32 cases of extragonadal and 12 cases of gonadal involvement. In Marina et al^[^^8^^]^, study within 73 patients with extra cranial immature teratomas, there were 51 gonadal involvements and 22 extra gonadal cases. In Brodeur GM et al^[^^9^^]^ study with the total number of57 patients, gonadal involvement was seen in 30 cases and extragonadal involvement in 27 cases.

**Stage at diagnosis: **In our study the most common stage at diagnosis was stage one (53%), and in Billmire et al. study^[^^1^^]^ with a total number of 26 cases they had 3 stage I to II, 5 stage III, and 17 stage IV. In another study by Billmire et al^[^^6^^]^, on 131 patients with primary ovarian tumor the most common stage was III (58 cases) followed by stage I (41 cases).

**Serum marker:** Mann et al^[^^10^^]^ evaluated 126 patients aged 0 to younger than 16 years with malignant GCT, serum AFP measured in 123 patients was elevated in 115 patients, whereas HCG was high in 19 of 77 cases. In our study only in 14 cases of yolk sac tumor and one patient of immature teratoma, AFP was elevated, and also BHCG was increased in one case of choriocarcinoma. Those patients in whom AFP level was very high (3 times more than upper limit of normal) died. As stated in the International Germ Cell Consensus Classification^[^^11^^]^ the degree of elevation of alpha-fetoprotein, human gonadotropin, and lactic dehydrogenase in adults is a risk factor for poor prognosis but this is not true in pediatric GCTs and only degree of elevation of AFP is associated with a poor prognosis in the pediatric population. This is consistent with the finding of our study.

**Survival: **The 5-year relative survival rate in Smith et al^[^^12^^]^ was 83.9%, and varied by histologic subtype, race, stage of disease, and age at diagnosis. The estimated 5-year survival rate in Marina et al^[^^13^^]^ is 100% for patients with stage I disease, 87% for stage II, 72% stage III, and 56% for stage IV. Six-year survival rate was 95% in stage I, 94% in stage II, 98% in stage III, and 93% in stage IV, in Billmire et al. study^[^^6^^]^. In Mann et al study^[^^14^^]^ the 5-year survival rate in all 184 patients was 93.2%. In our study follow up results demonstrated complete remission in 31 (77.5%), patients and 9 (22.5%) patients died.

## Conclusion

It seems that patients with extra-gonadal GCT and high AFP levels have a worse prognosis and lower survival rate; but a more powerful study including more patients from different pediatric surgery centers in Iran should be carried out in order to reach a solid conclusion. Combination of surgery and chemotherapy can lead to a better prognosis.
